# Exploring Disordered Regions of Human Spliceosome Proteins

**DOI:** 10.1021/acs.jpclett.6c00082

**Published:** 2026-02-14

**Authors:** Bruno de Paula Oliveira Santos, Krishnendu Bera, Luca Grisanti, Isabella Caterina Felli, Roberta Pierattelli, Alessandra Magistrato

**Affiliations:** † Department of Chemistry “Ugo Schiff” and Magnetic Resonance Center (CERM), 9300 University of Florence, via Luigi Sacconi, 6, 50019 Sesto Fiorentino (FI), Italy; ‡ CNR-IOM at SISSA, via Bonomea 265, 34136 Trieste, Italy

## Abstract

Introns are removed from mRNAs by the spliceosome, a type of protein–RNA machinery enriched with intrinsically disordered regions (IDRs). Lacking stable 3D structures, IDRs can adopt diverse conformations interlacing protein and RNA components of the spliceosome and regulating splicing. In this work, we performed a comprehensive bioinformatics analysis of the human spliceosome proteome, revealing that many proteins contain more than 40% disordered residues. Spliceosome IDRs are mainly driven by compositional bias due to an excess of charged and RS-like sequences, with the nature and extent of this disorder being broadly conserved evolutionarily. Additionally, these IDRs are frequent targets of post-translational modifications, especially phosphorylation, and are hot spots for cancer-associated mutations, which have been implicated in different types of cancer. Our results collectively underscore the central role of IDRs in splicing regulation and disease.

## Graphical abstract

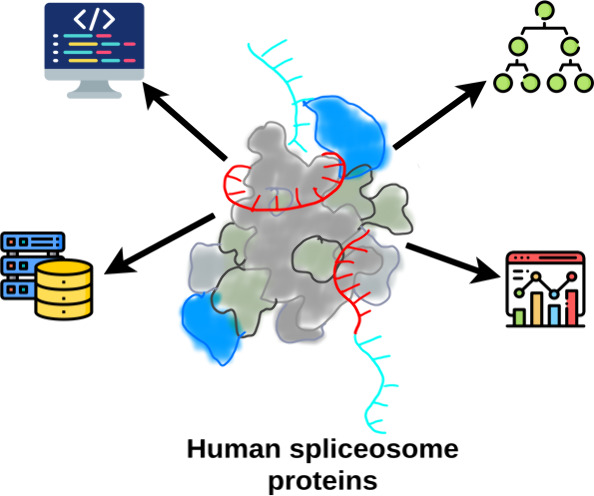

Intrinsically disordered proteins (IDPs) or proteins with intrinsically disordered regions (IDRs) lack regular three-dimensional structure and are characterized by a large set of dynamic and interconverting conformations. IDRs are widespread in the proteome and are present across all domains of life.^1^ They play critical roles in cellular functions such as transcriptional regulation, signal transduction, and subcellular organization. The plasticity and structural heterogeneity of IDRs indeed expand their repertoire of macromolecular interactions and allow them to be finely modulated by their structural and chemical environment.^2–4^ IDRs are also abundant in ribonucleoprotein complexes involved in gene expression, regulation, and synthesis.^5^ Among them, the spliceosome machinery, which promotes premature mRNA (pre-mRNA) splicing via dynamic protein/RNA binding and dissociation events, contains a large fraction of IDRs in its components.^6^

In eukaryotic cells, the spliceosome removes the noncoding regions (introns) from a pre-mRNA transcript and connects the coding sequences (exons).^7^ The most prevalent spliceosome form, the major spliceosome, consists of five small nuclear RNA (snRNA) strands (U1, U2, and U4–U6) and 100–200 associated proteins, which assemble into small nuclear ribonucleoprotein particles (snRNPs).^8,9^ The ordered and regulated assembly of these snRNPs and auxiliary proteins forms the spliceosome complex that undergoes extensive rearrangements during the splicing process. The resulting major spliceosome, which splices most introns (U2-dependent introns), uses the U1 and U2 snRNPs to scan pre-mRNA and identify specific intron sequences (splice sites). Namely, U1 and U2 snRNPs bind sequentially to the pre-mRNA, initially forming the E complex and later A complexes, where the initial recognition and assembly steps occur. In the subsequent steps of the cycle, the spliceosome remodels to perform catalysis, assembling into the B, B^act^, and B* complexes (the latter being where the branching reaction occurs).^10^ This is followed by the formation of the C and C* complexes (where the exon-ligation step occurs),^11^ the P complex, where the products are formed, and, finally, the intron lariat spliceosome (ILS) complex, where the intron and the exon are released^12^ and the spliceosome is dismantled to undergo a new splicing cycle.^4,13–15^ Besides those contained in snRNPs, additional proteins are involved in splicing. They can function individually or assemble into multiprotein auxiliary complexes, such as the nineteen complex (NTC) complex,^16^ the exon-junction complex (EJC),^17^ the cap-binding complex (CBC),^18^ the retention-and-splicing complex (RES),^19^ and the transcription–export complex (TREX).^20^

The second form of the spliceosome, the minor complex, splices a small class (<1%) of introns (U12-dependent introns), which have stronger splice site consensus sequences compared to U2-dependent introns. This spliceosome variant consists of four unique snRNAs (U11, U12, U4atac, and U6atac) and 14 unique protein factors.^21^ They work together with the core protein components shared by both spliceosomes likely through conserved mechanisms.

Irrespective of the spliceosome type, mutual recognition of spliceosome subunits, correct assembly, and component remodeling are paramount for splicing fidelity. In this context, IDRs play a key role in spliceosome function by forming weak interactions that interlace with many protein/RNA components and modulate binding dynamics. Moreover, IDRs can be finely regulated by post-translational modifications (PTMs), which readily alter their set of interactions.

Spliceosome IDRs can be divided into four categories: (i) regions containing predicted secondary structure (SS) elements (termed SS-IDR), (ii) long (≥25 residues) compositionally biased IDRs (termed CB-IDRs), which includes RS-like (IDRs rich in Arg and Ser), poly-P/Q (IDRs with repeats of proline or glutamine), and G-rich regions (IDRs with Gly repeats, RGG, [RSY]­GG, and R­[AGT]­[AGTFIVR]), with charged disorder and noncharged disorder,^5^ (iii) the IDR object of PTMs (PTM-IDR), and (iv) of cancer-associated mutations (CA-IDR).

To explore their abundance, types, and functional and regulatory roles, we performed an integrative sequence-based analysis of spliceosome proteins, focusing on their IDR content, complemented by statistical correlation analysis of PTM and cancer-associated sites, and phylogenetic inferences. Overall, this study deepens our understanding of IDRs in splicing, highlighting their implications in splicing regulation and disease.

To characterize the prevalence of intrinsic disorder in spliceosome proteins, we performed a bioinformatic analysis across different splicing steps, protein classes, and sequence contexts (Figures 1–3; see sections 1.1–1.4 of the Supporting Information for details). To this end, we retrieved the spliceosome sequences from the UniProt database (www.uniprot.org), predicted their disorder content, and calculated the disorder percentage in different groups.

**Figure 1:**
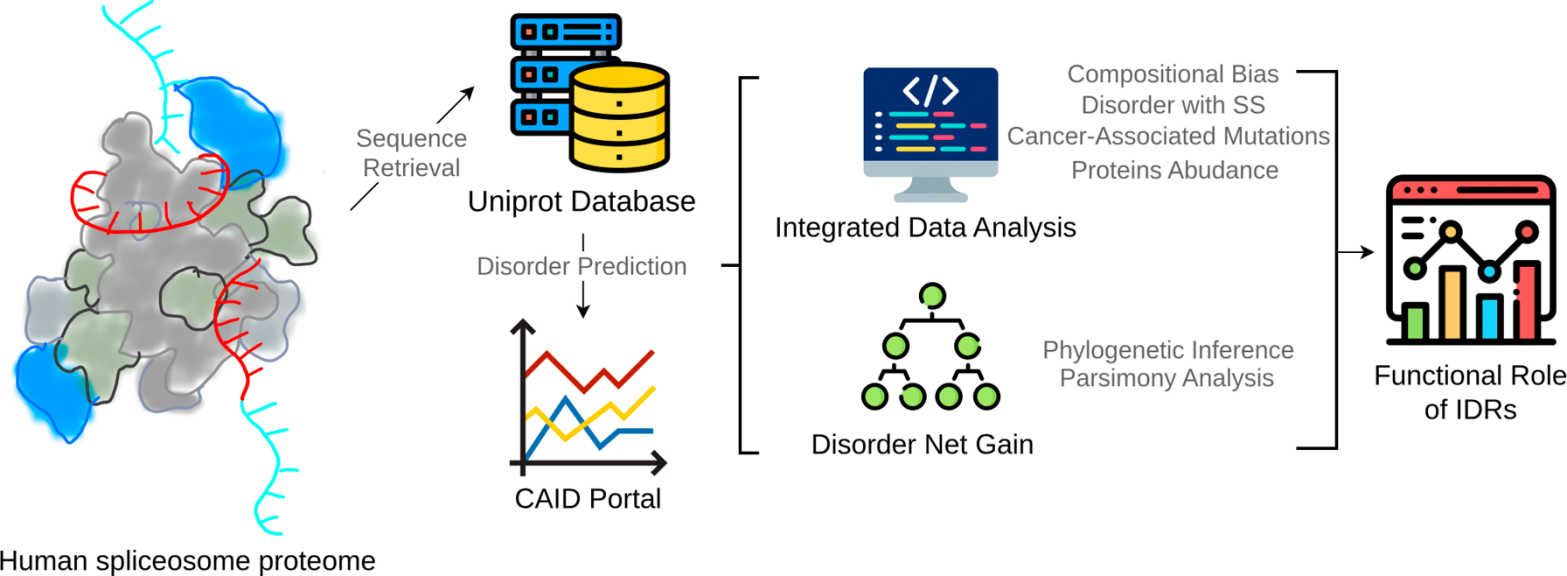
Pipeline of bioinformatic analysis of spliceosome IDRs. Bioinformatic analysis began with the retrieval of sequences from the UniProt database, followed by intrinsic disorder prediction. Sequence-based and statistical analyses were then performed to investigate the type of disorder (compositional bias or disorder with secondary structure) and the associated features, including post-translational modifications and cancer-associated mutations, and finally their evolutionary history.

We first evaluated the degree of disorder in the spliceosome complexes. We observed that in the major spliceosome the initial E complex exhibits the highest disorder content. The percentage decreases in the A complex until the pre-B complex is reached, slightly increasing in the B^act^ and B* complexes, where the first splicing reaction occurs, and remaining roughly constant until the P complex forms. The disorder content then decreases drastically at the ILS complex (Figure 2a). These data imply that in the central and final steps of the splicing cycles, where the spliceosome complex is fully assembled and engaged in catalysis and in dismantling its components, the plasticity of the IDRs is less crucial.

**Figure 2:**
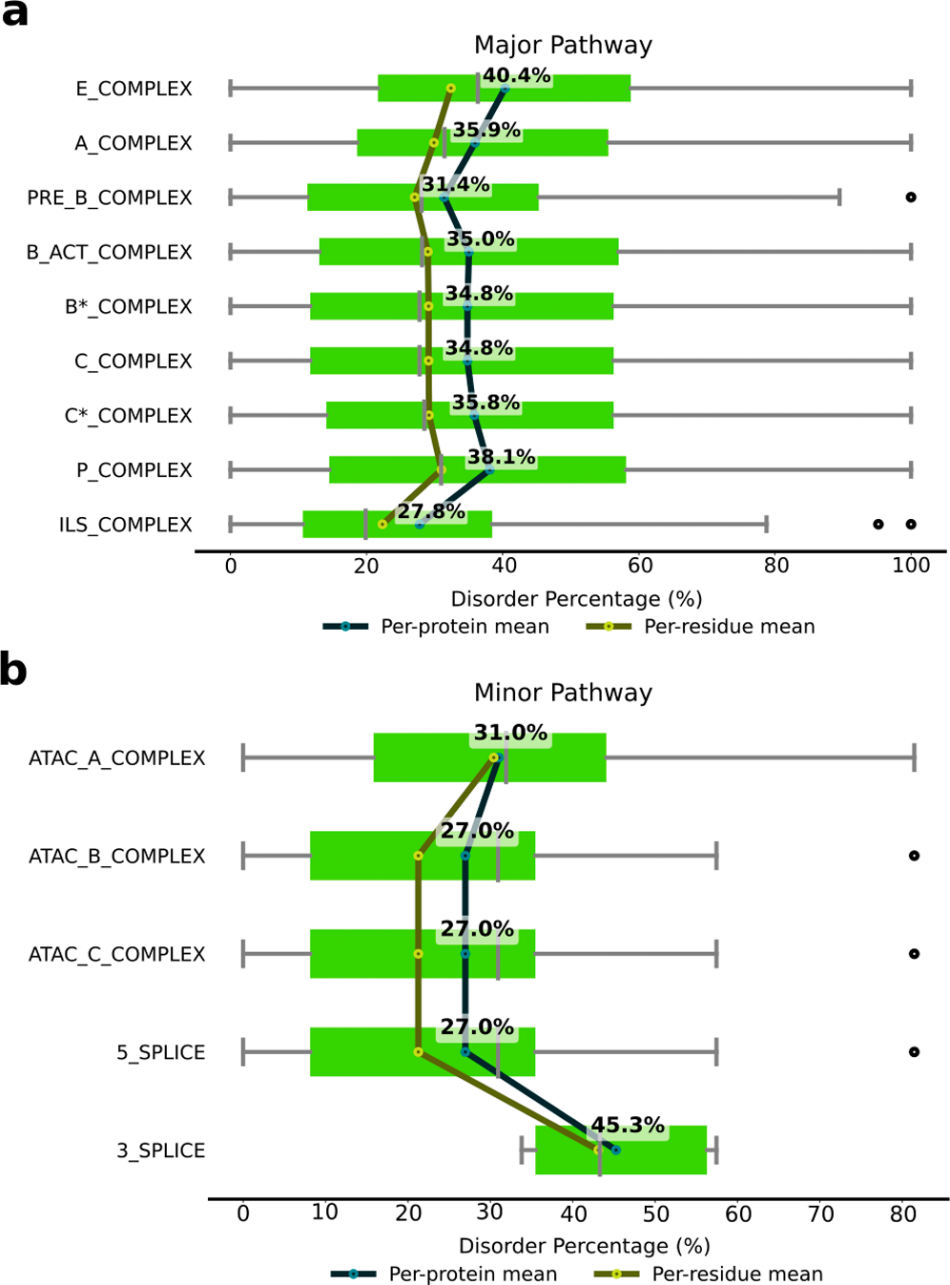
Disorder percentage in spliceosome complexes. Disorder content in complexes belonging to (a) major and (b) minor spliceosome pathways. The per-protein and per-residue means are shown as blue and yellow dots, respectively. The green bars are box plots, showing the distribution of disorder percentages with five key statistical measures: minimum value, first quartile (25th percentile), median, third quartile (percentile), and maximum value, along with outliers (black dots). The reported percentages refer to per-protein means, since they reflect the average disorder level of a typical protein within each class.

Although data on the minor spliceosome are more limited, it overall appears to contain a lower proportion of disordered residues, with the highest content being in the minor spliceosome A (AT-AC) complex (31%). Conversely, the disorder content of the remaining minor spliceosome specific complexes remains constant (27%) and increases during 3′-splice site cleavage (45.3%) (Figure 2b).

We next analyzed the disorder content by protein classes or families. We observed that only three families have less than 20% disorder content (i.e., “like Sm” (LSM, 10.8%), Gemin proteins (GEM, 15.2%), and TREX (19.4%)). Conversely, many families exhibited disorder content of at least 40% (i.e., proteins recruited at B^act^ complex (40.0%), nonspecified, i.e., no classes or families labeled/annotated (40.8%), U2 snRNP-associated (present in the A, B, B^act^, and B* complexes, 41.5%), recruited at B complex (44.8%), SR protein (47.6%), U1 snRNP (53.3%), both present in the E, A, and B complexes, and RES complex (present in the A, B, and B^act^ complexes, 70.8%)) (Figure 3a). These results confirm that the early spliceosome complexes contain proteins with the largest IDRs. However, the proportion of disordered and ordered sites is different in every complex (Figure 2a).

**Figure 3:**
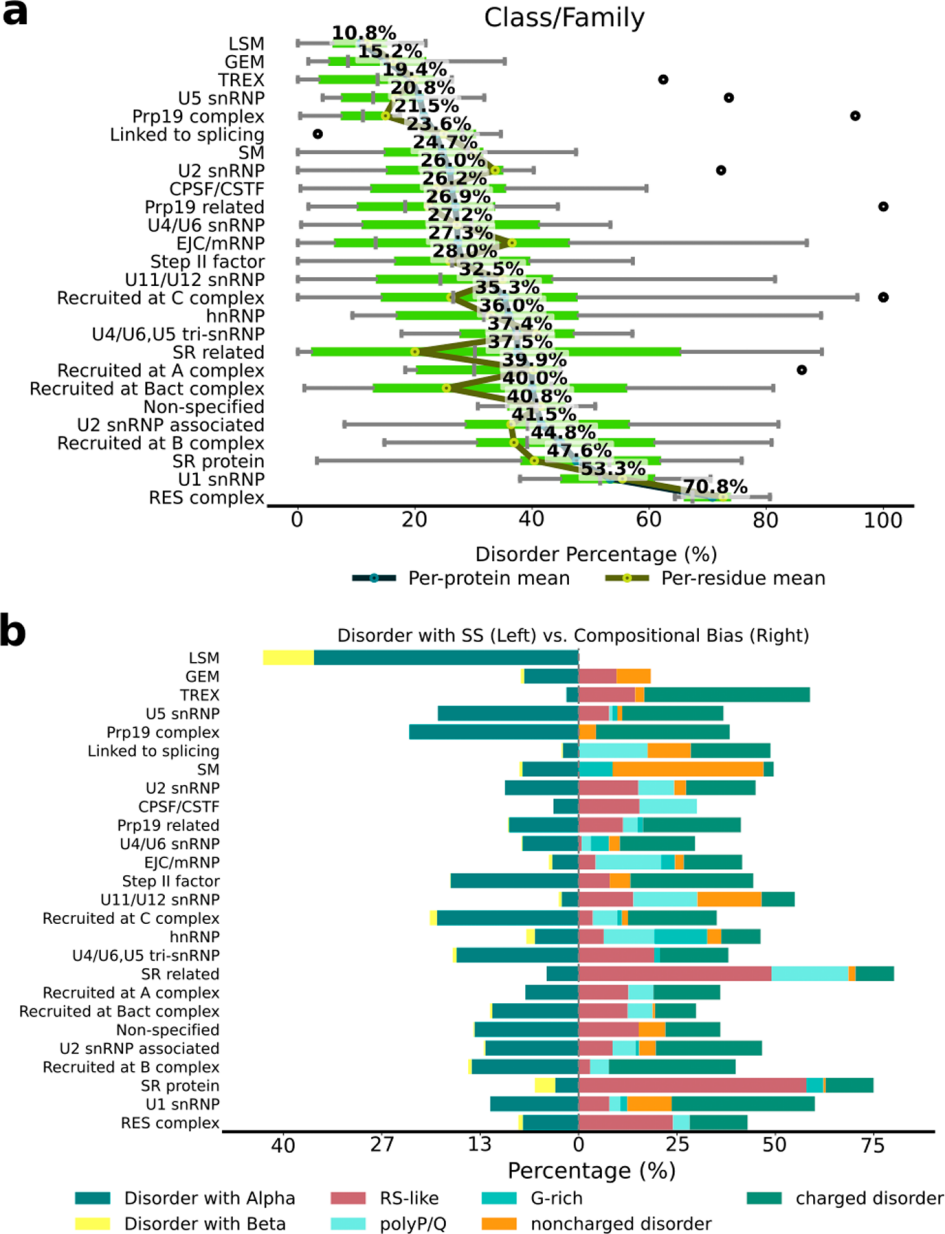
Disorder percentage and type of spliceosome. (a) Class/family of spliceosome proteins. Per-protein and per-residue means are shown as blue and yellow dots, respectively. The green bars are box plots, showing the distribution of disorder percentages with five key statistical measures: minimum value, first quartile (25th percentile), median, third quartile (75th percentile), and maximum value, along witg outliers (black dots). The reported percentages refer to per-protein means, reflecting the average disorder level of a typical protein within each class. (b) Type of intrinsically disordered content in different protein classes. The disorder content is divided into disorder with secondary structure (SS, left) and disorder with compositional bias (CB, right). For disorder with SS, the percentages of residues containing α-helix and β-sheet are colored yellow and blue, respectively. For disorder with CB, RS-like, poly-P/Q, G-rich, noncharged, and charged are colored red, light blue, cyan, orange, and dark green, respectively.

We also annotated the disorder types, classifying the IDRs as CB-IDR and SS-IDR. CB-IDRs are characterized by the presence of residues or motifs with a higher frequency than normally expected in the proteins of vertebrates (Figure 3b). This analysis revealed that the CB-IDR content varied between 0% and 75%, with the SR and SR-related proteins exhibiting the highest content.

The most common types of CB-IDR were associated with the presence of charged residues (18.57%), followed by RS-like motifs (12.82%) and poly-P/Q sequences (6.0%). However, the relative abundances of these CB-IDR segments were different in distinct protein classes (Table S1). Besides the RES, SR, and SR-like proteins, in which the RS-like content was the largest, in the other groups the CB-IDRs were owed to the presence of charged residues.

Conversely, the SS-IDR content, that are regions predicted to be disordered and to contain secondary structure content (i.e., α-helices or β-sheets) varied from 2% to 43%, with LSM (43.7%) being the class with the largest SS-IDR content, followed by the Prp19 complex (23.4%), proteins recruited at C complex (20.6%), and U5 snRNP proteins (19.5%). Within the SS-IDR, the α-helical type of secondary structure was predominant (Figure 3b and Figures S1 and S2).

Interestingly, the disorder content in the LSM, Prp19 complex, and U5 snRNP classes of proteins is low. The existence of these SS-IDRs in these regions suggests that they are transitional. In a few cases, IDRs were characterized by a proportional amount of CB-IDR and SS-IDR content (U4/U6.U5 snRNP, Step II factor, recruited at C complex, and Prp19 complex), suggesting that these classes may exhibit promiscuous behavior during splicing.

Next, we assessed the presence and type of PTMs occurring within the IDRs of spliceosome proteins (section 1.6 of the Supporting Information). First, we calculated the relative abundance of PTMs in ordered or disordered regions of the spliceosome proteins. An enrichment test, comparing the proportion of residues with PTMs in disordered regions versus ordered regions, showed that PTMs in IDRs are more abundant (Fisher test *p* value = 1.101 × 10^–115^). Namely, we identified 1012 sites hosting PTMs over 32 068 sites without PTMs in IDRs as compared to 831 sites hosting PTMs over 78 059 sites without PTMs in ordered regions.

Then, we calculated the density of PTMs per protein, revealing that PTMs per residue are significantly more abundant in IDRs (Wilcoxon test *p* value = 5.782 × 10^–15^). Additionally, we evaluated the PTM distribution per class/family (Figure 4a), confirming the prevalence of PTMs in IDRs (>50%). Finally, we inspected the relative abundance of each PTM type, defined as the proportion of PTMs located in IDRs relative to the total number of PTMs across the entire protein sequence (including both ordered and disordered regions). Interestingly, citrulline (0.66), phosphoserine (0.64), *N*-acetylalanine (0.6), and *N*-acetylserine (0.54) were more abundant in IDRs than in ordered regions (ratio of >0.5) (Figure 4b). Notably, these PTMs are commonly associated with diverse biological functions ranging from rapid signaling (phosphoserine) to epigenetic regulation (citrulline) and fundamental protein life-cycle management (N-acetylation),^22–26^ suggesting that they play similar regulatory roles in splicing.

**Figure 4:**
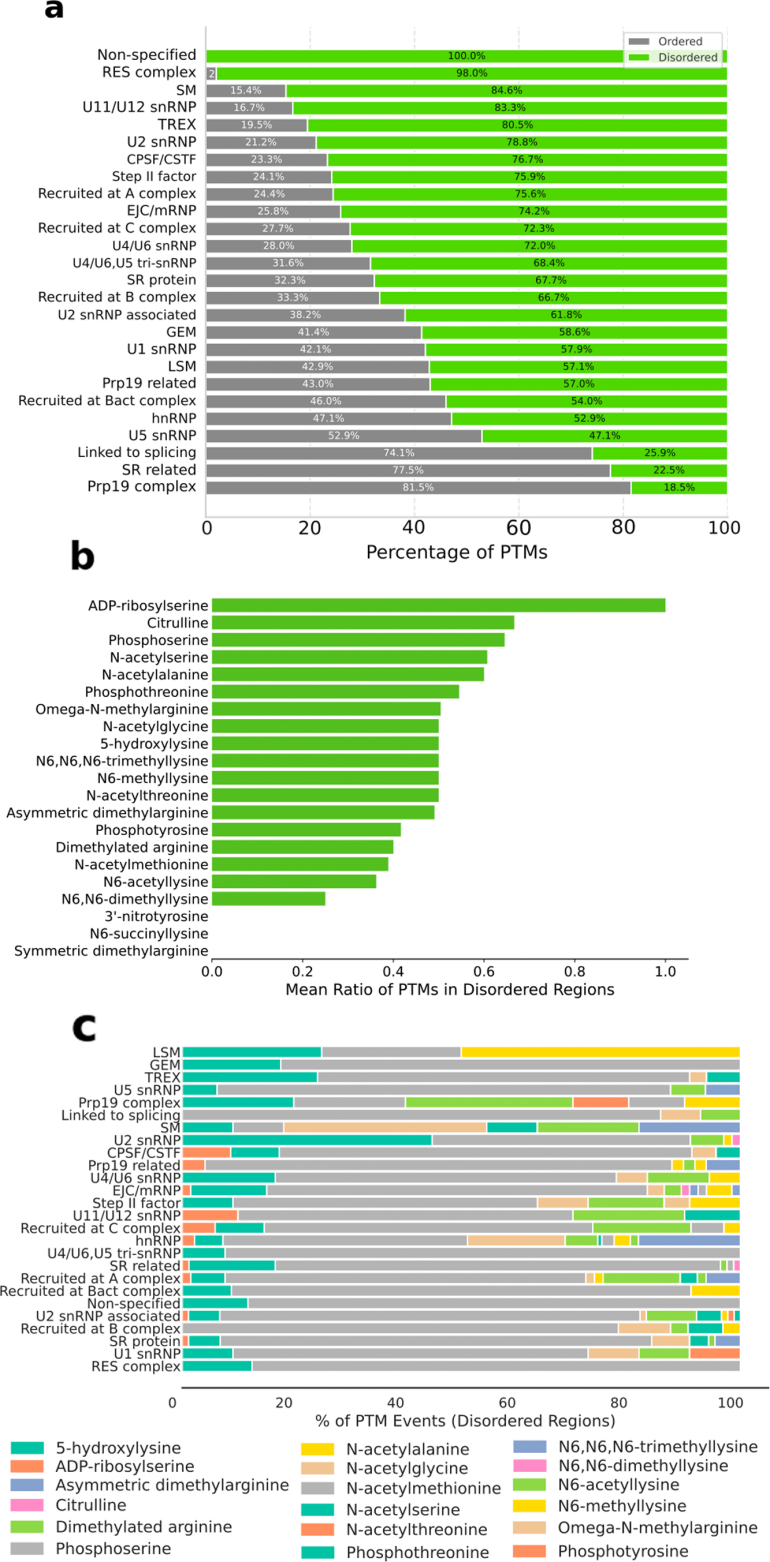
Distribution and characteristics of post-translational modifications (PTMs) in spliceosome proteins. (a) PTM distribution in different class/family groups (ordered vs disordered regions). (b) Comparison of the proportion of PTMs occurring within intrinsically disordered regions (IDRs) vs those found across the whole sequence of spliceosome proteins. (c) Relative abundance of different PTM types across groups/families of spliceosome proteins. Groups are listed according to PTM abundance from top to bottom. PTMs data, presented as percentages, provide insights into PTM preferences and potential functional specialization in subgroups.

Focusing on only IDRs, we then inspected the abundance of PTM types in different protein classes/families (Table S2), with phosphoserine (65,7%) and phosphothreonine (11.2%) being the predominant ones (the PTM percentage was calculated as the amount of a specific PTM type divided by the sum of the overall number of all PTM types) (Figure 4c). These PTMs may be involved in modulating protein–protein interactions and subcellular compartment regulation. Other PTM types, such as N6-acetyllysine (6.1%), ω-*N*-methylarginine (4.4%), *N*-acetylalanine (3.9%), and asymmetric dimethylarginine (2.4%), instead, showed class/family specific patterns.

Due to the centrality of the spliceosome for gene expression and regulation and its implication in cancer,^27–29^ we further investigated the frequency of cancer-causing mutations in IDRs (section 1.6 of the Supporting Information). This analysis revealed that among spliceosome protein families, the SR and hnRNP proteins are most frequently objects of mutations (Figure 4a). Overall, 43 proteins contained IDRs that host cancer-associated variants. Among them are FUS, RMB10, SF3B1, SRSF2, U2AF1, and THOC2 (Table S3). The number of mutations in the IDRs of these proteins varied from 1 to 5 and was associated with different types of tumors, with carcinomas being the most abundant category (Figure 5b). Remarkably, their implication in diverse cancer types reflects the broad impact of splicing alterations in tumorigenesis. Complete data on mutations in ordered and disordered regions are provided in Tables S3 and S4.

**Figure 5:**
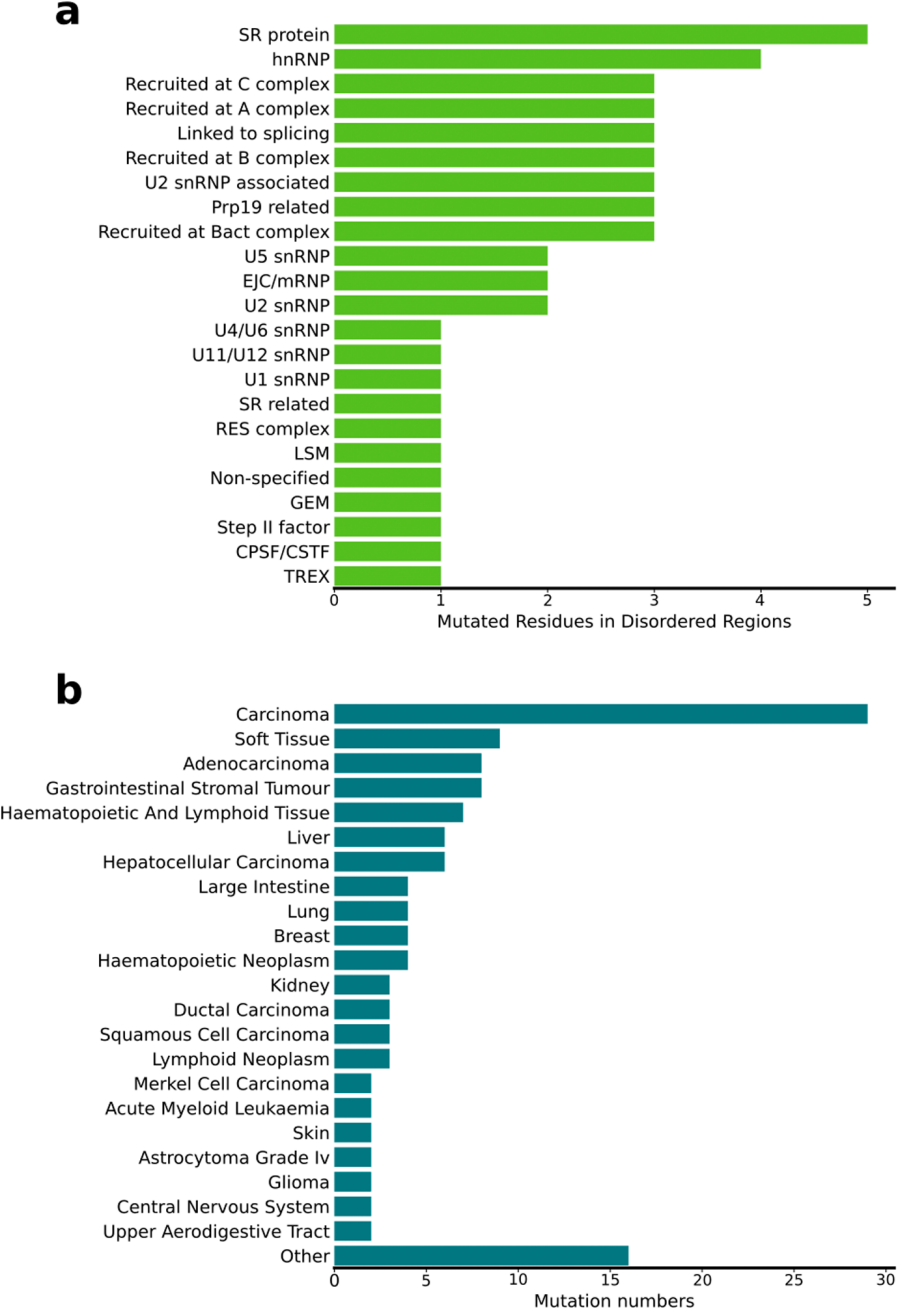
Cancer-associated mutations in intrinsically disordered regions (IDRs) of spliceosome proteins. (a) Number of mutated residues within IDRs across different protein families. (b) Number of mutations occurring in IDRs associated with distinct tumor types.

To check for an association between PTMs and cancer-associated variants, we even examined whether cancer-causing mutations, flanking (i.e., located within five residues^30^) the PTM site, occurred frequently.

This enrichment analysis identified 17 proteins hosting cancer-causing mutations near the PTM sites. In nine of these proteins (FUS, THOC2, SRSF2, WBP11, U2AF2, SF3B1, RBM10, DDX41, and RBM8A), these cancer-associated variants are related to skin abnormality (human phenotype ontology identifier HP:0000951), while in five of them (SRSF2, SF3B1, PRCC, DDX41, and RBM8A), they are related to neoplasms (HP:0002664). In both cases, the mutations are predominantly associated with phosphoserines and phosphothreonines (Table S5).

Next, we ranked the proteins by their disorder fraction and considered their cellular abundance. Among the proteins containing the largest IDRs, only a subset was highly abundant (Table 1). These proteins (SRRM1, FUS, YBOX1, and RU17) have disorder contents ranging from 50% to 90% and are predominantly characterized by a CB type of disorder, even if the type of residues causing the CB varies (Results 1 of the Supporting Information). The observed differences in the type of sequences causing the CB type of disorder may allow their multivalent interactions to be critical for the formation and remodeling of dynamic ribonucleoprotein complexes. Their high expression levels and disorder content suggest that these proteins may play critical roles in cellular homeostasis, as detailed in Results 1 of the Supporting Information. The regulatory role of FUS, SRRM1, and YBX1 is further supported by their hosting of multiple PTMs (Table S2).

**Table 1:** Spliceosome Proteins Exhibiting the Highest Disorder Fractions (more than 70%)

abundance	protein	disorder fraction	group	UniProt
abundant	SRRM1	89.49	SR-related	Q8IYB3
	FUS	89.35	hnRNP	P35637
	YBOX1	81.48	U11/U12 snRNP	P67809
	RU17	70.48	U1 snRNP	P08621
non-abundant	PQBP1	100	Prp19-related	O60828
	LENG1	100	recruited at C complex	Q96BZ8
	TLS1	100	recruited at C complex	Q9NZ63
	FA32A	95.54	recruited at C complex	Q9Y421
	CWC15	95.20	Prp19 complex	Q9P013
	CASC3	86.91	EJC/mRNP	O15234
	TR150	86.07	recruited at A complex	Q9Y2W1
	PKRI1	82.07	U2 snRNP-associated	Q9H875
	CWC25	81.18	recruited at B^act^ complex	Q9NXE8
	ZMAT2	80.91	recruited at B complex	Q96NC0
	BUD13	80.61	RES complex	Q9BRD0
	ZN830	78.76	recruited at B^act^ complex	Q96NB3
	SREK1	75.79	SR protein	Q8WXA9
	CD2B2	73.61	U5 snRNP	O95400
	RED	72.71	hnRNP	Q13123
	PPIG	72.28	U2 snRNP	Q13427
	SPF45	71.32	U2 snRNP-associated	Q96I25
	RNPS1	70.82	hnRNP	Q15287

Additionally, we investigated the evolutionary dynamics of spliceosome proteins exhibiting the highest IDR content (>70%) (section 1.5 and Results 2 of the Supporting Information and Figures S4–S25). Specifically, we inspected whether structural disorder played a role in the evolutionary history of these proteins and if their orthologs retained similar disorder content. To this end, we constructed phylogenies for 19 proteins. Multiple-sequence alignments revealed that, in most cases, the high disorder content was maintained across orthologs (Results 2 of the Supporting Information). In a few cases, specific clades deviated markedly, displaying loss of disorder, thus reflecting different evolutionary trajectories (Results 2 of the Supporting Information).

Commonly, disordered sites of proteins display rapid evolutionary dynamics, while ordered sites tend to be more conserved. This metric is evaluated through disorder-to-order transitions (DOTs), which refer to the change between ordered and disordered states. Interestingly, when analyzing this property in spliceosome IDRs, we observed that SS-IDRs, located adjacent to ordered regions in the same protein or at positions corresponding to ordered regions in ortholog proteins, exhibited significantly lower DOT rates compared to those of the other disordered regions (Mann–Whitney *U* = 2.39 × 10^6^; *p* < 10^–9^). Due to their lower DOT values and preservation of amino acid sequence, SS-IDRs appear to be more evolutionarily conserved than the other random disordered sites, suggesting that their secondary structure imposes evolutionary constraints across orthologs.

Notably, upon analysis of the increase or decrease in the disorder content across evolution (net gain of disorder), clades exhibited mixed trends, indicating protein specialization and different functions for the IDR content in different organisms (Figures S24 and S25). Collectively, this analysis revealed that the predominant evolutionary trend in IDRs was based on the persistence of SS-IDRs. These regions are thus likely conserved to play a functional role in splicing regulation.

We have finally estimated the liquid–liquid phase separation (LLPS) propensity of spliceosomal proteins using catGRANULE 2.0, a machine learning-based predictor.^31^ A large fraction of proteins displayed LLPS propensity scores of more than 0.5 (92%), with 67% being strongly LLPS-prone (>0.8), indicating enrichment in sequence features associated with phase separation (Table S6).

Previous computational studies indicated that intrinsic disorder is a defining feature of spliceosomal proteins. An initial study focused on serine/arginine-rich (SR) splicing factors, showing that SR proteins are enriched with disorder-promoting residues and lack stable folded structures.^32^ Subsequent bioinformatics analysis of the human and *Saccharomyces cerevisiae* spliceosomal proteomes revealed a marked enrichment of IDP/IDRs relative to their respective background proteomes.^33^ The spliceosome had an ordered catalytic core supported by peripheral, evolutionarily younger, IDR-rich proteins involved in early assembly, regulation, and dynamic remodeling.^34^ Computational identification of disorder-based binding sites with tools such as α-MoRFs and ANCHOR^35^ further supported the idea that intrinsic disorder facilitates spliceosome assembly, reversibility, and adaptability. As such, these studies established intrinsic disorder as a conserved, functionally essential property of the spliceosome and highlighted the central role of computational approaches in uncovering its structural and evolutionary principles.

Here we build and expand on previous findings predicting that half of the spliceosome proteins contain extensive IDRs (48.5%), which are unevenly distributed in amount and type. Notably, a large proportion (35.7%) of these proteins have >40% of their sequence classified as disordered. The disorder content is strongly correlated with RS-like and charged compositional bias, and approximately 15% of disordered regions alternate with secondary structure formation. The highest percentage of disorder is observed at the E complex, confirming that IDRs are key for early spliceosome assembly.

Notably, spliceosome IDRs are targets for PTMs,^36^ with phosphorylation of serine and threonine residues being the most abundant type. The addition of a bulky and negatively charged phosphoryl group is expected to regulate splicing by modulating interaction affinity or complex assembly, particularly in SR proteins and other splicing regulators.^37^ This regulatory principle is exemplified in several core spliceosome components. As an example, phosphorylation of SAP155 (U2 snRNP) is tightly coordinated with catalytic steps,^38,39^ phosphorylation of PRP28 by SRPK2 is essential for stable tri-snRNP integration,^40,41^ and multisite phosphorylation of SF3B1 N-terminal by CDK11 modulates RNA interaction within the B^act^ complex.^42^

Interestingly, only a few among the most disordered proteins (e.g., SRRM1, FUS, YBOX-1, and SNRNP70) are abundant in the human proteome. Their function may be that of interaction hubs within the spliceosome and in dynamic ribonucleoprotein aggregates. The remaining highly disordered proteins, characterized by lower cellular abundance, must instead be more specifically implicated in splicing regulation.

Across evolution, spliceosome proteins retain the SS-IDR content (Figures S24 and S25), which is conserved in different clades. In contrast to other protein families whose IDRs are rapidly evolving,^43^ spliceosome IDRs display low rates of disorder–order transitions throughout evolution, as reflected by their small number of changes per node (ranging from 0.01 to 0.08) . Notably, more changes per node are concentrated in SS-IDR segments, indicating that the limited DOT is focused on specific residue positions across lineages. This pattern suggests constrained flexibility, which is consistent with the function of spliceosome proteins as an essential type of cellular machinery evolving under purifying selection,^44^ and may be associated with the presence of phosphorylation sites.^45^

Interestingly, we identified multiple cancer-associated mutations in IDRs that cluster in splicing-related families, such as in SR proteins, in hnRNP, and mostly in proteins recruited at A, B, B^act^, and C complexes. Since spliceosome IDRs flexibly interlace proteins and RNA, even subtle perturbation of these interaction networks may alter their function, ultimately triggering splicing defects. The tumor types associated with these variants were diverse, confirming the systemic impact of splicing dysregulation in cancer.

Lastly, we predicted that splicesome proteins have high LLPS propensity, being strongly enriched with IDRs and containing RNA-binding domains.^46^ In a manner consistent with our prediction, several spliceosomal regulators and associated proteins were recurrently revealed to undergo liquid–liquid phase separation or to participate in phase-separated nuclear condensates. Among these, RBFOX1 and AKAP95 form dynamic assemblies mediated by low complexity or IDRs that contribute to splicing regulation.^47^ hnRNPs and serine/arginine-rich splicing factors, such as SRSF9, were observed to form condensate-like droplets with functional consequences for splice site usage and splicing regulation, while nuclear speckles (membraneless organelles enriched with spliceosomal components) exemplify how LLPS may organize the splicing machinery *in vivo*.^48^ Several spliceosome-associated proteins and RNA-binding proteins (RBPs), including core structural components of nuclear speckles like SON and SRRM2, contain extensive IDRs supporting multivalent interactions characteristic of phase-separated condensates.^49^ Spliceosome-associated factors, such as PLRG1, were shown to localize to nuclear speckles through LLPS-mediated interactions, facilitated by IDPs.^50^ In general, dynamic liquid-like nuclear condensates are enriched with RBPs and splicing factors and are thought to promote spliceosome assembly, spatial organization, and regulation of pre-mRNA splicing.^51^ Together, these observations support a model in which LLPS contributes to the functional organization and dynamic regulation of the spliceosomal machinery.

Overall, our study underscores the central role of IDRs in splicing regulation and disease, revealing that spliceosome IDRs are abundant, evolutionarily conserved, and functionally important regions that host regulatory and cancer-associated variants. These features align with the highly dynamic, transient protein–protein and protein–RNA interactions driving spliceosome function.

## Supplementary Material



## Data Availability

Data, scripts, and input are available at https://github.com/bposantos/spl_idp_project.git following the FAIR principles (10.1038/s41592-025-02635-0).
